# Increased CD8^dim^ and Decreased CD8^bright^ T Cells as Immunological Signature for Multibacilary Leprosy Patients

**DOI:** 10.1111/imm.13937

**Published:** 2025-05-05

**Authors:** Yuri Scheidegger de Castro, Letícia Silva Nascimento, Juliana Azevedo da Silva, Rebeka da Conceição Souza, Gabriel Nogueira Araújo, Sandra Chalhub de Oliveira, Edilbert Pellegrini Nahn Junior, Alba Lucínia Peixoto‐Rangel

**Affiliations:** ^1^ Laboratório de Biologia Do Reconhecer, Centro de Biociências e Biotecnologia, Universidade Estadual Do Norte Fluminense Darcy Ribeiro Campos dos Goytacazes City Brazil; ^2^ Hemocentro Regional de Campos dos Goytacazes Campos dos Goytacazes City Brazil; ^3^ Faculdade de Medicina de Campos Campos dos Goytacazes City Brazil

**Keywords:** CD8^bright^ and CD8^dim^ T cells, lymphocytes, *Mycobacterium leprae*

## Abstract

Leprosy, a chronic infectious disease caused by *Mycobacterium leprae* , manifests in a spectrum of clinical forms and severity. This study investigated the percentage of CD8^+^ T cells and their subpopulations (CD8^bright^ and CD8^dim^ T cells) in leprosy patients stratified by clinical forms, bacterial load, and age. No significant differences were observed in the overall percentage of CD8^+^ T cells among healthy controls and leprosy patients. However, an increased percentage of CD8^dim^ T cells and a decreased percentage of CD8^bright^ T cells were associated with severe multibacillary and lepromatous forms of leprosy, independent of bacillary load. Further, these cellular profiles correlated more strongly with disease severity than with age, in spite of elderly multibacillary patients exhibiting significant reductions in CD8^bright^ T cells and increases in CD8^dim^ T cells compared to young or middle‐aged paucibacillary patients, but not compared to young and middle‐aged multibacillary patients. These findings suggest that CD8^bright^ and CD8^dim^ T cell profiles are critical indicators of disease progression and severity in leprosy, highlighting their potential as biomarkers for clinical evaluation.

## Graphical abstract

Leprosy, a chronic infectious disease caused by *Mycobacterium leprae* , manifests in a spectrum of clinical forms and severity. This study investigated the percentage of CD8^+^ T cells and their subpopulations (CD8^bright^ and CD8^dim^ T cells) in leprosy patients stratified by clinical forms, bacterial load, and age. An increased percentage of CD8^dim^ T cells and a decreased percentage of CD8^bright^ T cells were associated with severe multibacillary and lepromatous forms of leprosy, independent of bacillary load and age of patients. These findings suggest CD8^bright^ and CD8^dim^ T cell profiles are critical indicators of severity in leprosy, highlighting their potential as biomarkers for clinical evaluation.

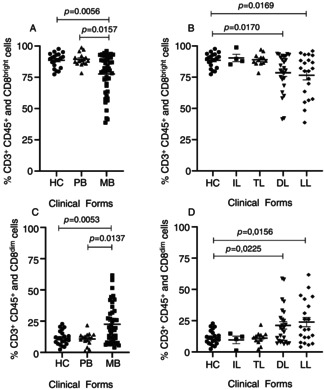

## 1 Introduction

Leprosy is a neglected chronic infectious disease caused by *Mycobacterium leprae* , which remains a public health problem in low‐income countries [1, 2]. *Mycobacterium leprae* mainly affects the skin and peripheral nervous system, causing loss of sensitivity, innervation, intraepidermal lesions, and lesions associated with loss of myelin in Schwann cells [3]. Around 95% of people are genetically resistant to *M. leprae* infection and 5% are susceptible, presenting a wide spectrum of clinical forms in agreement with the presence (or absence) of cell‐mediated immunity (CMI) against the pathogen.

The clinical spectrum is divided into poles, namely tuberculoid and lepromatous [1]. In tuberculoid leprosy, there is a polarisation of the immune response to Th1, an effective cellular response characterised by the production of IFN‐γ, which activates CD8^+^ T lymphocytes, macrophages, and bactericidal mechanisms that control the growth of *M. leprae* [4, 5]. On the other hand, the lepromatous pole exhibits a Th2 immune response with high production of IL‐4 and predominantly the activation of B lymphocytes, allowing the bacillus to evade the immune response, leading to a deficient protective response and, consequently, chronic infection [6, 7]. In the dimorphic form, patients display immunological and histopathological characteristics varying between tuberculoid and lepromatous forms, meaning that both Th1 and Th2 responses occur concomitantly [8]. Numerous leprosy classifications have been proposed since the 1930s, considering the two extremes of the spectrum as well as the intermediate (borderline) manifestations that lie between them. In terms of clinical characteristics, the Madrid classification from 1953 encompasses the tuberculoid (TT), lepromatous (LL), borderline, and indeterminate (IND) forms [9]. Leprosy is categorised as either paucibacillary (PB) or multibacillary (MB), depending on the number of skin lesions, the presence of nerve involvement, and the detection of bacilli in slit‐skin smears. Standard leprosy treatment typically entails the administration of multidrug therapy (MDT) [10].

CD8^+^ T cells play critical roles in fighting against intracellular pathogens, such as *M. leprae* , as well as eliminating malignant cells in cancer [11]. Upon antigen stimulation, naïve CD8^+^ T cells undergo robust expansion to give rise to effector and memory T cells. Effector CD8^+^ T cells, known as CD8^+^ CTLs, can directly induce target cell death by the interaction between Fas/Fas ligand and secretion of cytolytic mediator perforin, which creates pores in the target cells, allowing the delivery of granule serine proteases (granzymes) to induce apoptosis. Memory CD8^+^ T cells provide rapid and strong protection upon antigen reencounter, which is critical for effective and long‐term immunity. During CD8^+^ T cell differentiation, heterogeneous effector and memory populations have been identified, including short‐lived effector CD8^+^ T cells (TE), exhausted CD8^+^ T cells (Tex), long‐lived memory CD8^+^ T cells (TM), memory precursor CD8^+^ T cells (TMP), central and effector memory CD8^+^ T cells (TCM and TEM), and tissue‐resident memory (TRM) cells, which are named by their phenotype, differentiation potential and functionality [12, 13]. Of note, these subsets are produced at different time windows and tissue locations upon immune challenge, and their differentiation is under orchestrated regulation of TFs, epigenetic modification, and metabolic programs [14]. There are few reports in the literature about the role of CD8^+^ T lymphocytes in leprosy. The frequency of CD8^+^ T cells is similar among leprosy clinical forms, although functional features such as higher IL‐10 levels in MB compared to PB patients in this T cell subset have been observed [15]. Moreover, TNF‐α producing CD8^+^ T cells are essential in the pathogenesis of the type 2 leprosy reaction in MB patients. These reactions are episodes of acute hypersensitivity presenting as aggravation of the previous symptoms and skin lesions [16]. CD8^+^ T cells can be categorised into two major populations, CD8^bright^ and CD8^dim^ T cells, distinguished by their staining intensity with anti‐CD8 monoclonal antibodies that specifically recognise the CD8α chain [17]. CD8^bright^ T cells largely represent a CD3^+^ subset with MHC‐restricted cytolytic activity and suppressive effects on antibody production [18]. CD3^+^CD8^dim^ T cells are identified as a subset of CD8^+^ T cells with reduced CD8 expression. The CD8^dim^ T cells are thought to have impaired function and suppressive effects through various mechanisms in both humans and mice. The functional impairment of CD8^dim^ T cells is evident in their reduced capacity for proliferation and cytokine production. In HIV patients, naive CD8^dim^ T cells show defects in proliferation and TCR signalling, which are crucial for IL‐2 secretion; an increase in these cells is noted in cases of chronic and persistent antigen exposure, such as in HIV‐infected patients, transplant recipients, and mice chronically infected with *Trypanosoma cruzi* [18, 19, 20]. Given that distinct CD8^+^ T cell subpopulations, particularly CD8^bright^ and CD8^dim^ T cells, play crucial roles in the immunopathology of various infectious diseases by modulating immune responses and disease progression, we hypothesise whether these subpopulations could contribute to the pathogenesis of leprosy as well. So, our study investigated whether CD8^+^ fluorescence intensity on T cells was associated with leprosy severity, regardless of bacterial load or patient age.

## 2 Materials and Methods

### 2.1 Study Participants

Cases and healthy controls were recruited in Campos dos Goytacazes, Rio de Janeiro state, southeast Brazil (21°45′ 15″ S and 41°19′ 28″ W, 13 m asl). Sixty‐nine (69) leprosy patients were selected at the Hansen Health Program from Campos dos Goytacazes Health Secretariat, which is considered a reference center for treatment of this disease. All participants were clinically diagnosed according to the Brazilian's Ministry of Health Guidelines and the patient's diagnosis was confirmed by clinical examination and complemented by bacilloscopy of suspected tissue lesions. Peripheral blood samples were collected before treatment (Table 1). The present study enrolled 92 individuals with age range from 16 to 82 years old, 69 leprosy patients (LP) and 23 were healthy individuals (HC). Healthy controls were recruited from the local blood bank (hemocenter). Leprosy patients (LP) and healthy controls (HC) were from the same geographical area. Leprosy patients were grouped according to the World Health Organisation (WHO) classification in multibacillary (MB) or paucibacillary (PB) leprosy, and/or Madrid (1953) classification in lepromatous leprosy (LL), dimorph leprosy (DL), indeterminate leprosy (IL), and tuberculoid leprosy (TL) for the analysis (Table 1). Leprosy patients were also divided for age‐related analyses into elderly (*n* = 20), middle‐aged (*n* = 27) and young (*n* = 20). Exclusion criteria for participants were: pregnancy or breast‐feeding women and HIV. Hypertensive and diabetic individuals under drug control were included.

**TABLE 1:** Characteristics of study population.

	Clinical groups (WHO)/N (Age range)	Clinical groups (Madrid)	*N*	Age Mean ± SD (Range)	Gender
					Female/Male (%)
Leprosy (LP)	Multibacillary (MB) 53 (16–82)	Lepromatous (LL)	23	50.45 ± 15.44 (18–76)	8.69/91.31
		Dimorph (DL)	30	55.30 ± 18.45 (16–82)	20.00/80.00
	Paucibacillary (PB) 16 (18–65)	Indeterminate (IL)	4	37.75 ± 14.24 (18–50)	25.00/70.00
		Tuberculoid (TL)	12	39.91 ± 15.47 (19–65)	75.00/25.00
Total LP			69	50.32 ± 17.74 (18–82)	26.08/73.91
Healthy controls (HC)			23	36.00 ± 10.78 (20–54)	34.79/65.21

### 2.2 Ethics Statement

This study was performed according to the Helsinki Declaration. Written informed consent was obtained before enrolment. Patients received treatment according to national guidelines. Ethical approval of the study protocol was obtained through Faculdade de Medicina de Campos Ethical Research Committee (CAEE No. 19679119.8.0000.5244).

### 2.3 Cell Phenotyping and Flow Cytometry Analysis

**FIGURE 1:**
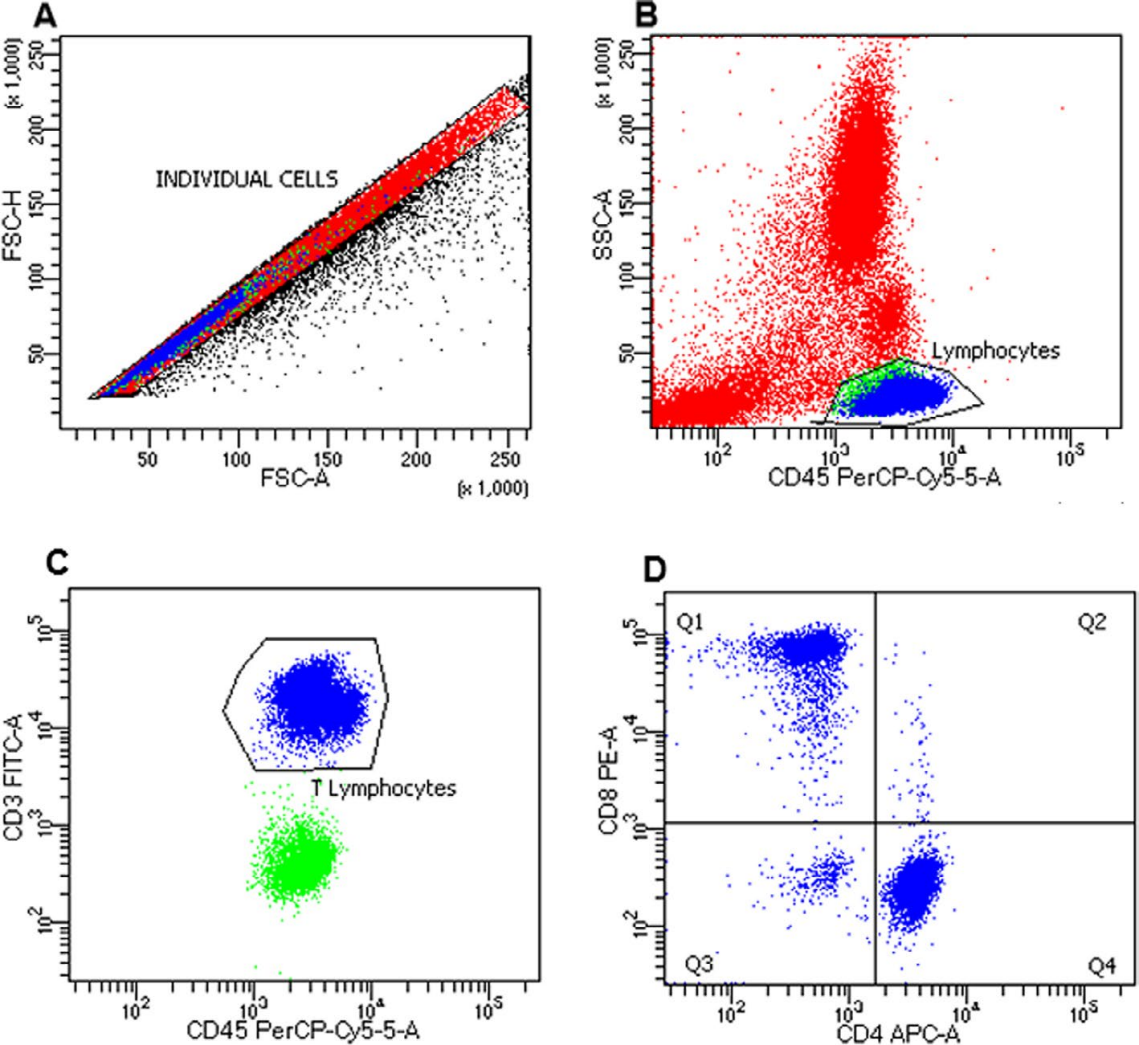
Gate strategy for CD8^+^ T cells in peripheral blood of leprosy subjects. (A) Representative flow diagram showing position of lymphocytes in forward (FSC) and side (SSC) scatter plot, (B) lymphocytes positive gate (CD45^+^ and SSC), (C) lymphocytes T positive (CD45^+^ and CD3^+^), (D) in quadrant 1 (Q1) representative of lymphocytes T CD8^+^.

Around 10 mL of peripheral blood samples from patients and healthy controls were obtained by venipuncture with vacuum tubes containing sodium heparin. Then, 25 μL of BD Multitest anti‐CD3 [fluorescein isothiocyanate (FITC)], anti‐CD8 [phycoerythrin (PE)], anti‐CD45 [Peridinin‐chlorophyll‐protein complex (PerCP)] and anti‐CD4 [allophycocyanin (APC)] fluorochromes‐conjugated monoclonal antibody was added to 50 μL of blood sample in a 1 mL tube. The tube was vortexed gently and incubated for 15 min in the dark at room temperature (20° to 25°C). Next, 450 μL of 1x FACS Lysing Solution (BD Biosciences) was added and vortexed gently, then incubated for 15 to 30 min in the dark at room temperature. After that, 500 μL of 1X FACS Lysing Solution was added to the tubes and subjected to centrifugation at 500×g for 10 min at 4°C. Samples were acquired on BD FACSymphony A1 with a 4‐laser configuration (BD Biosciences) and analysed using BD FACSDiva, version 9.0.2 (BD Bioscience, NJ, USA). The phenotyping of the cells is shown in Figure 1 and displays a gate strategy.

### 2.4 Statistical Analysis

Differences in the percentage of CD8^+^ T cells were statistically analysed using the nonparametric test Kruskal‐Wallis, followed by Dunn's test (posttest), for comparisons among three or more groups. The data are represented as medians ± standard error, and statistical analyses were performed using GraphPad Prism version 8.0 Software (GraphPad Software, La Jolla, CA), considering a significance level of 5%.

The influence of bacterial load on the percentages of CD8^bright^ and CD8^dim^ T cells was verified through the Ordinary Least Squares (OLS) regression analysis using the stats models. The dependent variables in this test were the CD8^bright/dim^ T cells, with the independent variable “BAAR” (alcohol‐acid resistant bacilli status encoded as integers: 0 for negative, 1 for +, 2 for ++, and 3 for +++) and considering R^2^ closer to zero and *p* value greater than 0.05, without correlation.

Non‐parametric correlation analysis, scipy.stats.spearmanr function, from the SciPy library in Python was used for pairwise comparisons between the CD8^bright^ and CD8^dim^ T cells count and Age for each categorical variable PB vs. MB. Spearman correlation coefficients (r) closer to zero and corresponding *p*‐values greater than 0.05 indicate no associations between variables.

## 3 Results

### 3.1 Ex Vivo CD8 ^+^ T Cells Do Not Differ Between Leprosy Patients and Healthy Controls

**FIGURE 2:**
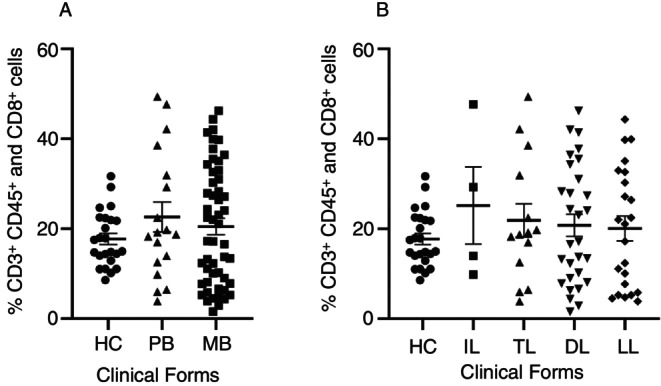
Percentage of CD8^+^ T cell population in different clinical forms and healthy control. (A) WHO classification: MB, Multibacillary; PB, Paucibacillary; HC, Healthy control and Kruskal‐Wallis statistical test, followed by Dunn's; (B) Madrid Classification: Clinical forms DL, Dimorphous; HC, Healthy control; IL, Indeterminate; LL, Lepromatous; TL, Tuberculoid. The data are represented as means ± standard error of mean (SEM) and analysed using Kruskal‐Wallis statistical test, followed by Dunn's.

To evaluate whether the percentage of CD8^+^ T cells was different among distinctive clinical forms of leprosy and HC, patients were grouped in WHO classification (Figure 2A) and Madrid (Figure 2B). There were no statistical differences between the percentage of CD8^+^ T cells among healthy and leprosy groups (Figure 2).

### 3.2 Increased CD8^dim^ and Decreased CD8^bright^ T Cells Profile Are Associated With Severe Leprosy but Not With Bacillary Load and Age

**FIGURE 3:**
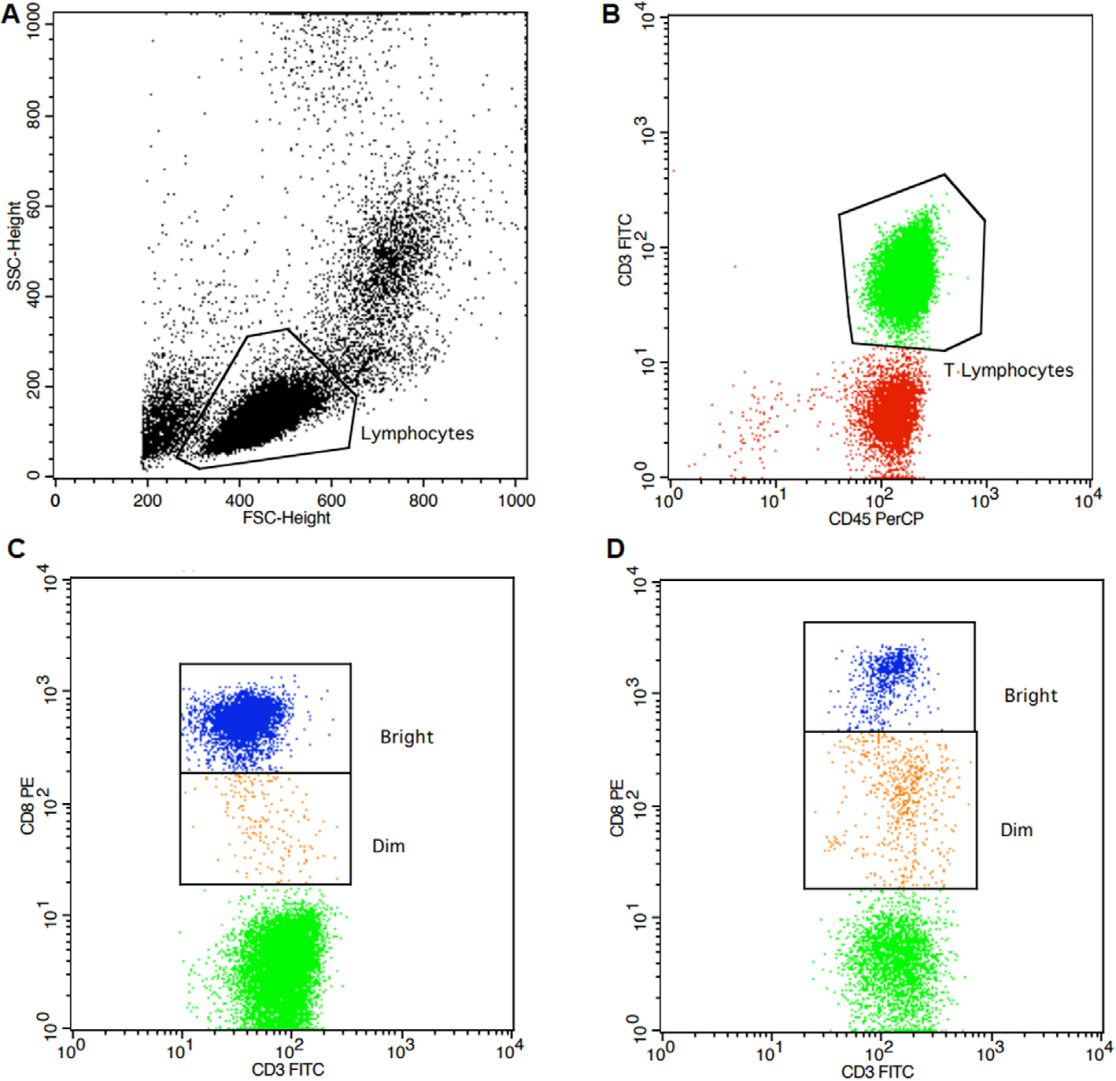
Gate strategy for CD8 bright and CD8 dim T cells in peripheral blood of leprosy subjects. (A) Representative flow diagram showing position of lymphocyte in forward (FSC) and side (SSC) scatter plot, (B) lymphocytes positive gate (CD45^+^ and CD3^+^), (C and D) lymphocytes positive gate to CD8^dim^ and CD8^bright^ T cells (CD3^+^ and CD8^+^), that represent tuberculoid and lepromatous forms respectively.

Two subpopulations with distinct fluorescence intensity of the receptor CD8 on these T cells, named CD8^bright^ and CD8^dim^ T cells, were observed (Figure 3).

**FIGURE 4:**
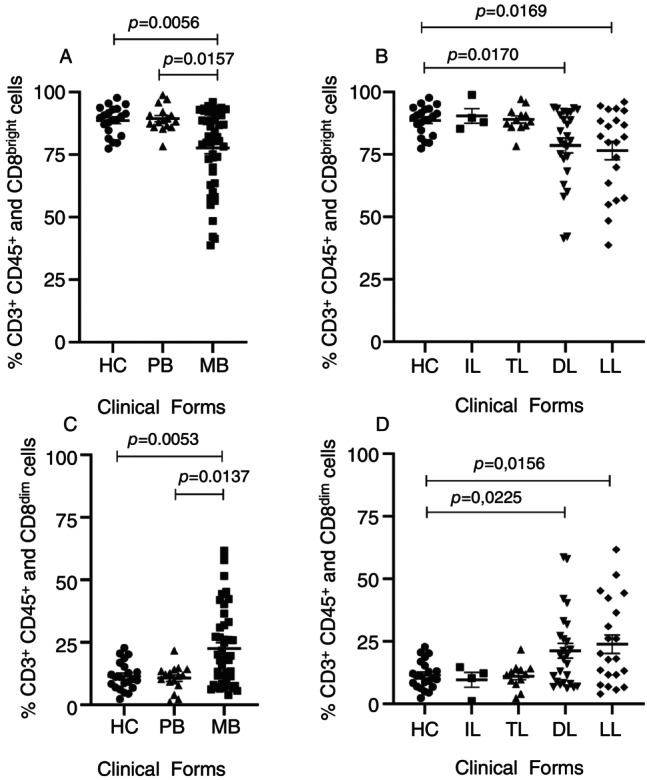
Percentage of the median CD8^bright^ and CD8^dim^ T cells in leprosy. (A and C) WHO classification: HC, Healthy Control; MB, Multibacillary; PB, Paucibacillary; (B and D) Madrid classification: Clinical forms DL, Dimorphous leprosy; HC, Healthy Control; IL, Indeterminate leprosy; LL, Lepromatous leprosy; TL, Tuberculoid leprosy. The data are represented as means ± standard error of mean (SEM).

We observed CD8^bright^ T cells percentage had a significant reduction in multibacillary (MB) compared to paucibacillary (PB) forms (*p* = 0.0157) and HC (*p* = 0.0056) (Figure 4A). As well as lepromatous leprosy (LL) (*p* = 0.0169) and dimorphic leprosy (DL) (*p* = 0.0170) compared to HC (Figure 4B). On the other hand, we observed an increased percentage of CD8^dim^ T cells in multibacillary compared to paucibacillary (*p* = 0.0137) forms and HC (*p* = 0.0053) (Figure 4C). The percentage of CD8^dim^ T cells also was higher in leprosy patients with the most severe forms of leprosy, DL (*p* = 0.0225) and LL (*p* = 0.0156), compared to HC (Figure 4D). The data indicate an association of increased CD8^dim^ and decreased CD8^bright^ T cells with severe form of leprosy.

**FIGURE 5:**
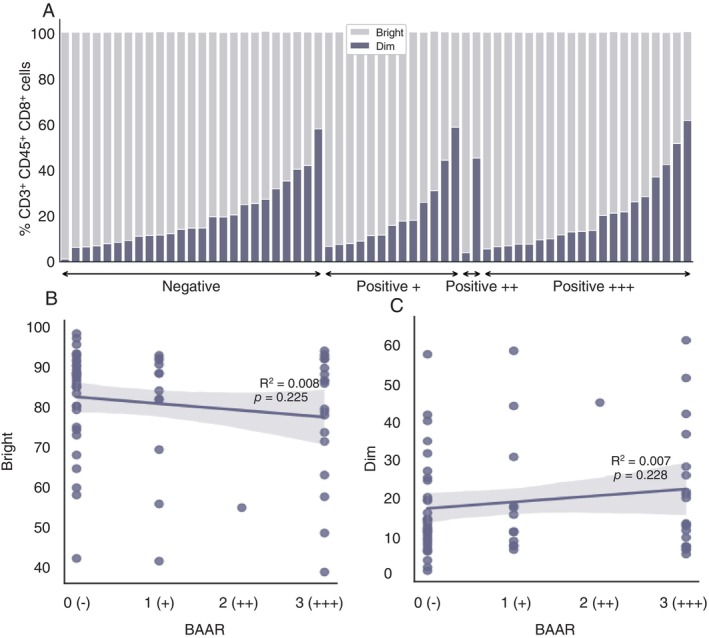
Relationship between percentages of CD8^bright/dim^ T cells and bacterial load in leprosy patients. (A and B) Scatter plots of BAAR vs. CD8^bright/dim^ T cells in Leprosy disease. This figure presents scatter plots with regression lines with R squared (R^2^) and *p*‐values showing the relationship between “BAAR” and the cellular counts. (A) R^2^ = 0.092 and *p* = 0.947 for CD8^bright^ T cells; (B) R^2^ = 0.092 and *p* = 0.936 in CD8^dim^ T cells, both indicating non‐significant associations. (C) Leprosy patients were grouped in negative (tuberculoid and indeterminate), and positive (dimorphic and lepromatous) with + (from 3 to 9 bacilli on the entire smear slide), ++ (≥ 10 bacilli on the entire smear slide), and +++ (≥ 10 per smear field).

And the variation in fluorescence intensity of the receptor CD8 on T cells was not correlated with the bacillary load obtained from bacilloscopy, since negative paucibacillary patients had a higher percentage of CD8^bright^ T cells, but some patients had an increased percentage of CD8^dim^ T cells. Similarly, multibacillary patients exhibited high levels of both CD8^bright^ and CD8^dim^ T cells (Figure 5A **)**. Because there are paucibacillary and multibacillary patients with high levels of both CD8^bright^ and CD8^dim^ T cells, these data indicate that there is no clear relationship between the levels of these CD8 subpopulations and bacillary load. To prove this statement, we performed a linear regression test and observed, indeed, there is no association between CD8^bright^ [R^2^ = 0.008; *p* = 0.225; CI (−4.4378 to 1.0658)] CD8^dim^ [R^2^ = 0.007; *p* = 0.228; CI (−1.0787 to 4.4378)] T cells and bacillary load, respectively (Figure 5B,C).

**FIGURE 6:**
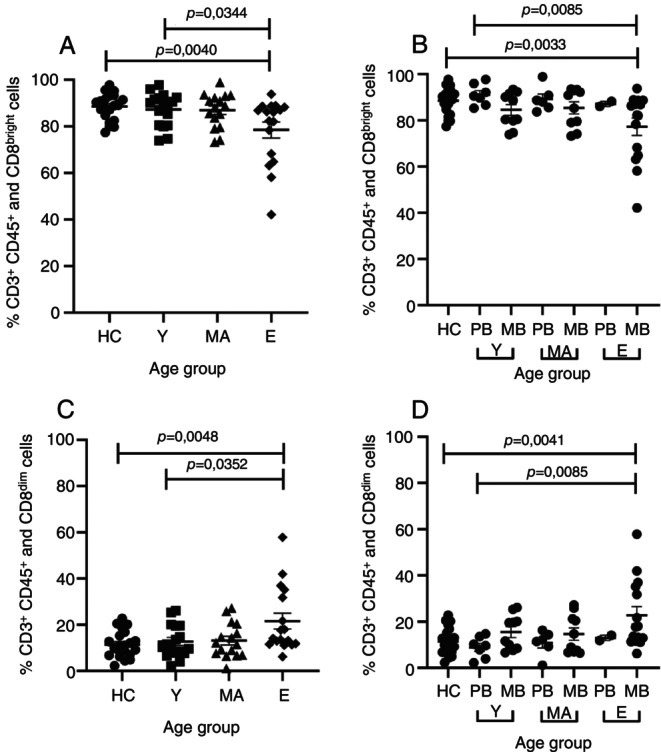
Percentage of the median CD8^bright/dim^ T cells in leprosy patients stratified into three groups of different ages. HC, Healthy control and the patients were categorised into three age groups: Y, young (18–40 years old); MA, middle‐aged (41–60 years old), and E, elderly (over 60 years old). (A and C) Categorical classification in patients with the different forms of leprosy. (B and D) WHO classification: MB, Multibacillary; PB, Paucibacillary; HC, Healthy Control and Kruskal‐Wallis statistical test, followed by Dunn's. The data are represented as means ± standard error of mean (SEM) and analysed using Kruskal‐Wallis statistical test, followed by Dunn's.

Since the bacterial load was not related to the fluorescence intensity of the CD8 receptor, we evaluated whether age could be associated with this variable in leprosy patients and healthy controls. So, we separated the study participants into the following groups: elderly (E) over 61 years old, middle age (MA) from 41 to 60 years old, young (Y) from 16 to 40 years old, and healthy controls (HC) from 20 to 54 years old. Elderly leprosy patients presented a significant reduction of CD8^bright^ T cells compared to the healthy controls (*p* = 0.0344) and young leprosy patients (*p* = 0.0040) (Figure 6A). In order to verify whether this reduction was related to age or disease severity, leprosy patients were separated into PB and MB according to age ranges for young, middle age, and elderly. Elderly patients with multibacillary forms (MB) had a CD8^bright^ T cells reduction when compared to HC (*p* = 0.0033) and the young with PB forms (*p* = 0.0085), but not when compared to elderly patients with PB forms (Figure 6B). Conversely, CD8^dim^ cells were increased in elderly patients compared to HC (*p* = 0.0048) and young patients (*p* = 0.0352) (Figure 6C). Similarly to CD8^bright^ T cells, this increase was related to elderly patients with MB forms when compared to the HC (*p* = 0.0041) and the young PB forms (*p* = 0.0085) (Figure 6D), but not when compared to elderly patients with PB forms. These data suggest that disease severity exerts more influence on CD8^bright^ and CD8^dim^ T cell levels than patient age.

**FIGURE 7:**
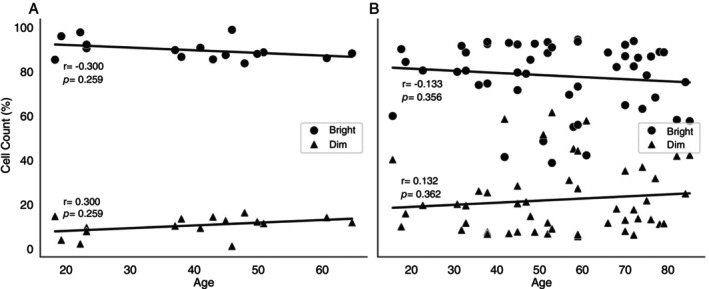
Scatter Plots of Age vs. CD8^bright/dim^ T cells for PB and MB patients in Leprosy disease. (A and B) Displays scatter plots with nonparametric Spearman correlation and *p* values for “Age” versus “CD8^bright/dim^ T cells”. (A) PB patients for CD8^bright^ (r = −0.300, *p* = 0.259) and CD8^dim^ (*r* = 0.300, *p* = 0.259) T cells. (B) MB patients for CD8^bright^ (r = −0.133, *p* = 0.356) and CD8^dim^ (*r* = 0.132, *p* = 0.362) T cells. The data suggest weak or negative correlations between age and cellular counts for PB and MB diseases.

In order to verify whether age really had a lesser impact on the levels of CD8^bright^ and CD8^dim^ T cells than the disease severity, we applied a correlation test considering the variables age and cells for PB patients (Figure 7A) and MB patients (Figure 7B). The data confirmed there were no correlation between age and CD8^bright^ and CD8^dim^ T cells, suggesting only the severity of the leprosy is associated with the reduced percentage of CD8^bright^ and increased CD8^dim^ T cells.

## 4 Discussion

The subsets of CD8^bright^ and CD8^dim^ T cells are two subpopulations of CD8^+^ T lymphocytes reported with distinct functional activities for other diseases [21, 22]. CD8^bright^ T cells largely comprise a CD3^+^ subset with cytolytic activity restricted to MHC and suppressive activity for antibody production. In contrast, T cells expressing low levels of the CD8 receptor (CD8^dim^) have been previously observed in persistent viral infections, and these cells have been characterised by inefficient cytotoxic activity [21, 22, 23, 24, 25, 26, 27]. However, in leprosy, the profile of CD8^bright^ and CD8^dim^ T cells is still not described.

In the present study, we also observed these two subpopulations of CD8^+^ T cells in leprosy patients, but their percentage seemed to vary among clinical forms of leprosy. So, we hypothesised CD8^dim^ T cells could drive the severity spectrum of the disease, since the percentage of these cells appears increased in multibacillary cases (Figure 4). On the other hand, CD8^bright^ T cells were reported to present a higher capacity to control HIV viremia compared to CD8^dim^ T cells [28]. This may suggest that a similar situation could occur in leprosy, since these cells are of higher frequency in the tuberculoid pole (Figure 4). Additionally, CD8^dim^ T cells were associated with the HIV viral load, meaning that a higher viral load corresponded to an increased quantity of these cells [28]. Subpopulations of CD8^dim^ T cells have also been documented to play a role in mechanisms likely to inhibit the cellular immune response or in the evasive strategies of microorganisms during the infection by feline immunodeficiency virus (FIV) [29]. Another study has shown CD8^dim^ T cells in the infection by sporotrichosis in cats, where the elevated levels of these cells were correlated with a higher fungal load, also indicating that this low fluorescence expression is associated with a lack of control over sporotrichosis and the severity of the clinical condition [30, 31]. Another example is the increase in circulating CD8^dim^ T cells among chronic HBV patients correlated with the progression of the disease. Patients with more extended disease duration tended to exhibit a higher frequency of CD8^dim^ T cells [27]. However, in our data there was no correlation with bacterial load, since some patients from both poles of the disease had a high frequency of the CD8^dim^ T cells, even though they had a low bacterial load (Figure 5). So, we believe that these cells may be influenced by other immunological mechanisms, such as persistence of antigen, the individual's age, and/or natural cell senescence in response to *M. leprae* infection.

CD8^dim^ T cells exhibit reduced antigen sensitivity and possess the ability to inhibit the responsiveness of their CD8^bright^ counterparts, potentially achieved through the production of TGF‐β [32]. In the study by Clutton and collaborators, CD8^dim^ T cells were associated with an increased frequency of signs of senescence (CD57) and mitochondrial alterations in individuals affected by HIV under antiretroviral therapy [32]. Our data indicate that the expanded population of CD8^dim^ T cells is associated with the MB pole and elderly patients, and then, the advanced age could have the same impact that the most severe clinical form of leprosy has on the CD8^bright^ and CD8^dim^ T cells. However, when we evaluated the correlation between age and the number of CD8^bright^ and CD8^dim^ T cells (Figure 7), we observed a weak correlation and without statistical significance, where PB patients for CD8^bright^ presented r = −0.300/ *p* = 0.259 and CD8^dim^ presented *r* = 0.300/*p* = 0.259. While MB patients for CD8^bright^ had *r* = −0.133/*p* = 0.356 and CD8^dim^ had r = 0.132/*p* = 0.362. This supports the result from Figure 6, which only elderly MB showed increased CD8^dim^ T cells and decreased CD8^bright^ T cells in relation to healthy and PB Young groups. Thus, the clinical form seems to exert a greater influence on the TCD8 subpopulations than age.

Additional studies should be carried out to evaluate the senescence of these cells, considering the same groups studied in this work, in relation to cytokine production and cytolytic capacity of CD8^dim^ and CD8^bright^ T cells. The aging process leads to innate and adaptive immune system adaptations, which result in increased susceptibility to infections, reduction in vaccine response, and even greater incidence of cancer [33]. Herein, two CD8^+^ T cell subsets, which differ from the median intensity fluorescence in relation to the CD8 marker, were shown in different percentages in elderly and young groups in leprosy patients, but the severity of the disease is the main influence on CD8^bright^ and CD8^dim^ percentages. Considering that *M. leprae* is capable of infecting a large amount of people, and only a minority will progress toward the disease [1], factors behind delayed disease onset that could be associated with changes in T lymphocyte subpopulations remain unclear. T‐cell exhaustion is a phenomenon of dysfunction or physical elimination of antigen‐specific T cells reported in human immunodeficiency virus (HIV), hepatitis B virus (HBV), and hepatitis C virus (HCV) infections, as well as cancer [34, 35].

Exhaustion appears to be often restricted to CD8+ T cell responses in the literature, although CD4+ T cells have also been reported to be functionally exhausted in certain chronic infections [36]. Although the understanding of the molecular mechanisms associated with the transcriptional regulation of T cell exhaustion is advancing, it is imperative to also explore it in the context of leprosy. It could be better explained this imbalance of CD8^dim^ and CD8^bright^ T cells between young and elderly groups with severe and mild leprosy.

## 5 Conclusion

Our findings reveal an immunological signature in multibacillary leprosy patients, characterised by an increased percentage of CD8^dim^ T cells and a decreased frequency of CD8^bright^ T cells. While the overall proportion of CD8^+^ T cells remains unchanged between leprosy patients and healthy controls, the altered distribution of these subpopulations suggests a role in disease severity. Importantly, this imbalance was not associated with bacterial load, indicating that other immunological mechanisms such as antigen persistence or immune senescence may drive these changes. Additionally, although elderly patients exhibited similar trends, statistical analyses suggest that disease severity exerts a stronger influence on CD8+ T cell subpopulations than age. These findings provide new insights into the immune dysregulation in leprosy and highlight the need for further studies to assess the functional properties of CD8^bright^ and CD8^dim^ T cells, including their cytotoxic activity and cytokine production, to better understand their contributions to disease progression and potential therapeutic strategies.

## Author Contributions

All authors were involved in drafting the article or revising it critically for important intellectual content, and all authors approved the final version to be published. Dr. Alba L. Peixoto‐Rangel had full access to all data in the study and took responsibility for the integrity of the data and the accuracy of the data analysis. Study conception and design: Alba L. Peixoto‐Rangel. Acquisition of data: Yuri Scheidegger de Castro, Letícia Silva Nascimento, Edilbert Pellegrini Nahn Junior, Sandra Chalhub de Oliveira. Analysis and interpretation of data: Yuri Scheidegger de Castro, Juliana Azevedo da Silva, Rebeka da Conceição Souza, Gabriel Araújo, and Alba L. Peixoto‐Rangel.

## Conflicts of Interest

The authors declare no conflicts of interest.

## Data Availability

The data that support the findings of this study are available from the corresponding author upon reasonable request.
